# Physics-Informed Neural Networks for Modeling Postprandial Plasma Amino Acids Kinetics in Pigs

**DOI:** 10.3390/ani16040634

**Published:** 2026-02-16

**Authors:** Zhangcheng Li, Jincheng Wen, Zixiang Ren, Zhihong Sun, Yetong Xu, Weizhong Sun, Jiaman Pang, Zhiru Tang

**Affiliations:** Laboratory for Bio-Feed and Molecular Nutrition, College of Animal Science and Technology, Southwest University, Chongqing 400715, China; lzhangcheng@163.com (Z.L.); 19123128428@163.com (J.W.); rzx2282003081@outlook.com (Z.R.); sunzh2002cn@aliyun.com (Z.S.); xuyetong@swu.edu.cn (Y.X.); swz2012@swu.edu.cn (W.S.); pangjm@swu.edu.cn (J.P.)

**Keywords:** physics-informed neural networks, non-linear least squares, postprandial amino acids kinetics

## Abstract

Postprandial plasma amino acid (AA) kinetics serve as essential indicators of digestive efficiency and systemic metabolic status in pigs. Traditional kinetic analysis relies on Non-Linear Least Squares (NLS) regression using compartmental models, yet these methods typically demand repeated blood sampling and precise initialization to ensure convergence. In this study, we developed a Physics-Informed Neural Network (PINN) framework by integrating mechanistic Ordinary Differential Equations (ODEs) directly into the deep learning loss function. The framework was evaluated using a benchmark dataset. Specifically, we performed a retrospective analysis by downsampling the original high-frequency data to simulate dense and sparse sampling strategies. The results demonstrate that while both models exhibit high fidelity under dense sampling, PINN maintains superior robustness and predictive accuracy under data-constrained conditions. Under the sparse sampling scenario, PINN reduced the Root Mean Square Error (RMSE) compared to NLS in key metabolic profiles, such as Methionine in the FAA group (*p* < 0.01) and Lysine in the HYD group (*p* < 0.05). Unlike NLS, which is sensitive to initial guesses, PINN successfully utilized physical laws as a regularization term to robustly solve the inverse problem, demonstrating superior parameter identification stability and predictive consistency under data-constrained conditions compared to NLS. We concluded that the PINN framework provides a reliable and consistent alternative for modeling the AA dynamics. In the future, it may be possible to reconstruct highly accurate physiological trajectories under optimized sparse sampling conditions.

## Simple Summary

Understanding the dynamics of amino acid absorption in pigs is crucial for optimizing animal nutrition and growth efficiency. However, traditional mathematical models used to analyze these digestive processes often struggle when data is scarce, requiring researchers to perform frequent and invasive blood sampling to ensure accurate results. This study aimed to overcome these limitations by developing a Physics-Informed Neural Network, an advanced artificial intelligence approach that integrates established biological laws of digestion into the learning process. We compared this new method against standard techniques using data from pigs fed various protein diets. Our results demonstrated that the PINN-based model was significantly more robust, maintaining high predictive accuracy even when the number of blood samples was drastically reduced. Unlike traditional methods, it successfully reconstructed complete digestive profiles from sparse data without requiring manual adjustments. We concluded that this technology offers an alternative for nutritional modeling. By adopting this method, high-precision analysis can be achieved with minimal samples in future, reducing the frequency of blood sampling while ensuring data quality.

## 1. Introduction

Postprandial plasma amino acid (AA) dynamics serve as critical indicators of digestive efficiency, absorptive capacity, and the systemic metabolic status of pigs. Dietary protein is hydrolyzed by proteases and peptidases in the stomach and small intestine into free AAs and small peptides. These products are actively absorbed by the epithelial cells of the small intestinal mucosa, subsequently transported via carriers to the capillaries, and finally enter the bloodstream through the portal vein [1]. This process is typically characterized by an asymmetrical bell-shaped plasma AA concentration curve. van Milgen et al. [2] proposed a biological concept-based Non-Linear Least Squares (NLS) regression model, utilizing a modified Erlang function to characterize the postprandial dynamic changes of plasma nutrient concentrations in pigs. This model simplifies complex, asymmetrical bell-shaped concentration curves into four core parameters: the basal fasting target concentration (*C_target_*), the meal-induced change in target concentration (*C_δ_*), the total metabolic exposure (*AUC*), and the response rate constant (*λ*). Validated through histidine deficiency trials, the model not only accurately fits concentration data across varying AA balance states but also demonstrates a linear correspondence between dietary supply and metabolic exposure. This provides a standardized tool for the quantitative assessment of postprandial nutrient metabolism from a biological perspective. However, They are highly sensitive to initial parameter guesses and typically require repeated blood sampling to ensure numerical convergence. Such intensive requirements may increase sampling burden. When researchers are constrained by sparse data to reduce the logistical burden and potential physiological interference, traditional regression often fails to reach stable solutions or produces biased estimates that lack mechanistic reliability.

To bridge the gap between complex biological mechanisms and limited experimental observations, Physics-Informed Neural Networks (PINNs) have emerged as a transformative deep learning paradigm. Unlike purely data-driven “black-box” models that may yield physically implausible predictions, PINNs embed mechanistic Ordinary Differential Equations (ODEs) directly into the neural network’s loss function. By leveraging automatic differentiation, the PINN architecture acts as a “physiological anchor,” ensuring that the predicted AA trajectories strictly adhere to the governing laws of mass balance and metabolic homeostasis. This synergy allows for robust parameter identification and continuous trajectory reconstruction, even under conditions of high experimental noise or reduced sampling frequency.

The objective of this study was to develop and validate a PINN-based framework for modeling postprandial AA kinetics in pigs. We integrated the van Milgen mechanistic ODEs into the PINN architecture and evaluated its performance using a comprehensive multisubject benchmark dataset [3] encompassing diverse physiological stages and dietary treatments. By benchmarking the PINN against traditional NLS methods under both dense and sparse sampling conditions, we aimed to demonstrate the model’s superiority and its potential to reduce the experimental and handling burden in porcine nutritional studies.

## 2. Materials and Methods

### 2.1. Data Source and Partitioning Strategy

The experimental data utilized in this study were sourced from the open-access benchmark study published by Eugenio et al. [3] This reference dataset characterizes the postprandial plasma AA kinetics in multicatheterized pigs, encompassing distinct physiological stages (growing and adult pigs) and dietary treatments. The original repeated blood sampling protocol (t = 0, 10, 20, 30, 40, 50, 60, 75, 90, 105, 120, 150, 180, 210, 240, 360 min) provides a high-resolution physiological ground truth. The dataset comprises three distinct dietary treatments characterized by different protein forms but identical AA profiles:

Intact Protein (INT): A diet containing feather meal as the sole protein source, representing a slow-release nutrient profile.

Hydrolyzed Protein (HYD): A diet containing extensively hydrolyzed feather meal (providing 82% free AAs and 18% small peptides), representing a rapid-release profile.

Free Amino Acids (FAA): A synthetic diet formulated with free crystalline AAs to mimic the AA profile of the HYD diet.

We selected eight essential amino acids (EAAs) for the fitting comparison.

### 2.2. The Mechanistic Kinetic Model

We adopted the mechanistic framework proposed by van Milgen et al. [1] as the physical constraint for the neural network. The governing Ordinary Differential Equations (ODEs) derived from the modified Erlang function are as follows:

(1) $$\left\{\begin{array}{l} \frac{dX_{1}}{dt}=-\lambda X_{1}\;\\ \;\frac{dX_{2}}{dt}=\lambda X_{1}-\lambda X_{2}\;\\ \;\frac{dC}{dt}=\lambda X_{2}-\lambda \;(C-\;(C_{target}+C_{\delta })) \end{array}\right.$$

where *X*_1_ and *X*_2_ represent the amount of nutrient in the proximal and distal transit compartments (e.g., gastric and intestinal phases), respectively. *C*_(*t*)_ represents the plasma concentration. The PINN is trained to minimize both the data discrepancy and the physical residual of these ODEs, thereby learning the key physiological parameters: *λ* (fractional flow rate), *C_target_* (basal concentration), and *C_δ_* (metabolic setpoint adaptation).

### 2.3. Physics-Informed Neural Networks

**Figure 1:**
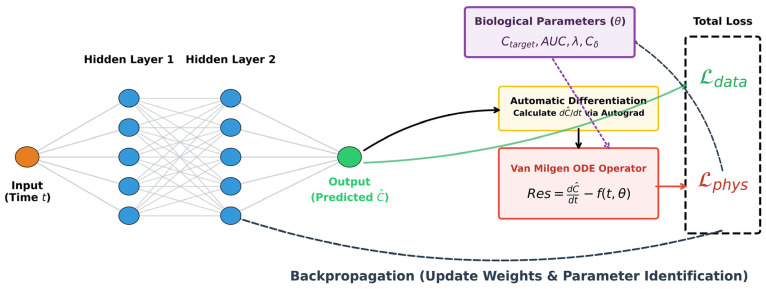
Architecture of the PINN for modeling postprandial AAs kinetics. The framework consists of a surrogate neural network to approximate AA concentration ($\hat{C}$) and a mechanistic branch that computes the physical residual based on the van Milgen ODE. Biological parameters (*θ*) are identified through the joint minimization of data fidelity loss ($L_{data}$) and physiological consistency loss ($L_{phys}$).

The integrated architecture of the proposed PINN, which couples the deep learning surrogate with the mechanistic ODE, is illustrated in Figure 1.

To optimize the parameter estimation process, we represent the concentration field using a feed-forward neural network N parameterized by weights W and biases b. The network takes time *t* as input and outputs the predicted concentration $\hat{C}$:

(2) $$\hat{C}\left(t\right)=N\left(t;W,b\right)$$

The core of the PINN approach is the integration of the biological ODEs into the neural network’s loss function. We define a total loss function L_tot_ that encourages the network to respect both the experimental measurements and the underlying physiology:

(3) $$L_{tot}=L_{data}+\omega_{p}L_{phys}\;$$

The individual loss components are defined as follows:

(1) Data Loss:

(4) $$L_{data=}\frac{1}{N}\sum_{i=1}^{N}{∥\hat{C}\left(t_{i}\right)-C_{obs,i}∥}^{2}$$

(2) Physics Loss:

(5) $$L_{phys}={∥\frac{d\hat{C}}{dt}-\left[f_{Erlang}(t,AUC,\lambda )-\lambda (\hat{C}-C_{target_{-}curr})\right]∥}_{\Omega }^{2}\;$$

### 2.4. Training and Optimization

To ensure robust convergence, the biological parameters *θ* = {*C_target_*, *AUC*, *λ*, *C_δ_*} are treated as trainable variables alongside the network weights. Training is conducted in two stages: an initial exploration phase using the Adam optimizer, followed by a refinement phase using the Limited-Memory Broyden–Fletcher–Goldfarb–Shanno (L-BFGS) Optimizer to achieve high-precision parameter identification. All inputs are normalized to the range of [0, 1] to improve gradient stability.

### 2.5. In Silico Simulation of Sparse Sampling Strategy

Instead of random sub-sampling, we designed a physiologically-guided sampling protocol to validate the model’s efficiency. Based on established kinetic profiles [2,4,5,6], the absorption peak of crystalline AAs typically occurs within 30–90 min. Therefore, we strategically retained sampling points at 0, 30, 60, and 90 min to capture the high-dynamic absorption phase, while widening the intervals during the elimination phase (150, 240, 360 min). This represents a resource-optimized strategy rather than random data loss.

### 2.6. Baseline Methodology and Performance Evaluation

A traditional NLS regression approach was employed as a performance benchmark. The NLS fitting was performed using the Levenberg–Marquardt algorithm to solve the same mechanistic ODEs described in Section 2.2. The predictive accuracy of both models was quantified using the Coefficient of the Root Mean Square Error (RMSE):

(6) $$RMSE=\sqrt{\frac{1}{n}\sum_{i=1}^{n}\;(y_{i}-{\hat{y}}_{i}{)}^{2}}\;$$

A paired *t*-test was used for mean differences, and *p* < 0.05 indicated that the difference was statistically significant.

### 2.7. Initialization Sensitivity Analysis

To systematically evaluate the robustness of the proposed framework against parameter uncertainty, we conducted a quantitative sensitivity analysis focusing on the dependency of model convergence on initial parameter guesses. Since traditional NLS methods are theoretically sensitive to the convexity of the solution space, we tested whether the physics-informed regularization in PINN could expand the basin of attraction for the optimization process. For each dataset, the “ground truth” reference parameters (*θ_ref_*) were first established using the best-fit results from the dense sampling data. We then introduced a controlled perturbation factor *δ* to generate biased initial guesses (*θ_init_*), defined as:

$$\theta_{init}=\theta_{ref}\times (1+\delta )$$

where *δ* represents the deviation percentage, ranging from −50% to +50%, with a step size of 2.5%. Both the NLS and PINN models were initialized using these perturbed parameters (*θ_init_*) and optimized under identical conditions. The final convergence stability was assessed by calculating the RMSE between the reconstructed trajectory and the ground truth data. A model was considered to have “diverged” if the final RMSE exceeded a threshold of 30% of the mean concentration, indicating a failure to recover the true physiological kinetics from the biased initialization.

## 3. Results

### 3.1. Model Verification and Baseline Accuracy on Dense Data

To establish a baseline for model performance, both PINN and NLS frameworks were evaluated using the full-resolution dataset (16 time points, n = 8).

**Figure 2:**
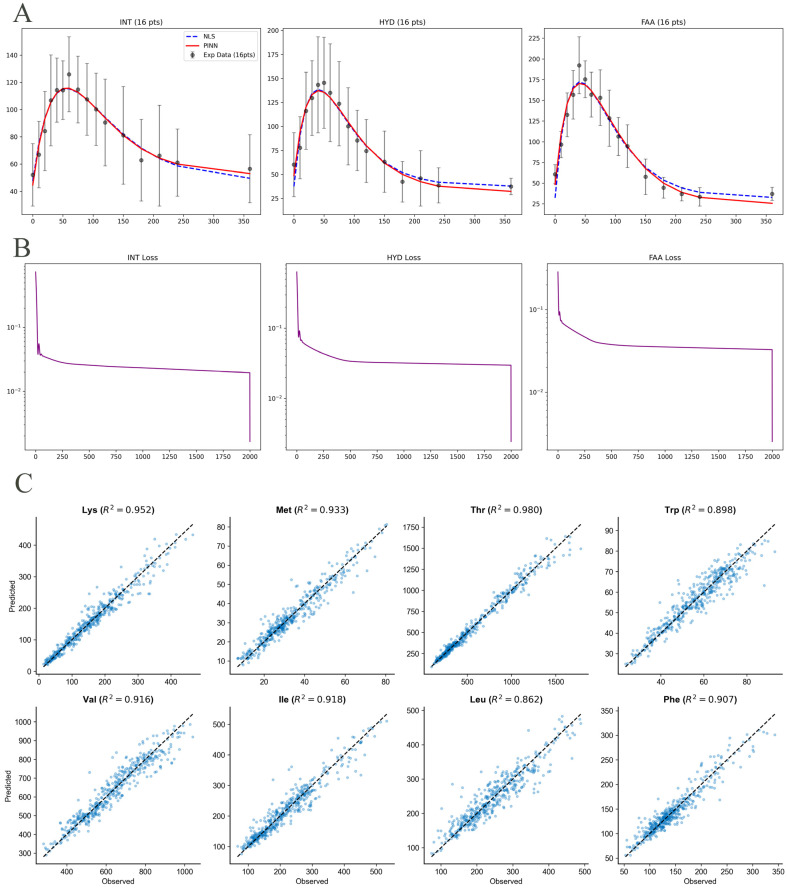
Baseline performance evaluation of the PINN framework on the n = 8 training dataset (dense sampling). The evaluation covers eight EAAs. (**A**) Taking Lys as an example, we show a comparison of observed data (black dots), traditional NLS fits (blue dashed lines), and PINN predictions (red solid lines). (**B**) Goodness-of-Fit Analysis: Scatter plot of predicted versus observed plasma concentrations. (**C**) Quantification of Reconstruction Error: Bar chart showing the RMSE for PINN and NLS methods under non-sparse conditions.

As illustrated in Figure 2A, both the PINN (red solid lines) and NLS (blue dashed lines) models demonstrated exceptional fidelity in capturing the postprandial kinetic profiles of Lysine (Lys) across all three dietary treatments (INT, HYD, and FAA). Under the full-sampling scenario, the two models produced nearly identical trajectories, accurately identifying the initial baseline, the rapid absorption peak, and the subsequent homeostatic clearance phase. The seamless alignment of PINN with the high-frequency experimental data confirms its reliability as a high-fidelity tool for AA kinetic modeling.

The training dynamics of the PINN framework are characterized by the loss convergence curve in Figure 2B. The total loss, which combines data-driven residuals and physics-informed constraints, exhibited a sharp exponential decay during the initial 2000 epochs of Adam optimization, followed by high-precision fine-tuning using the L-BFGS algorithm. The stable convergence to a global minimum without obvious oscillations suggests that the hybrid optimization strategy effectively balances the empirical data fitting with the mechanistic requirements of the van Milgen model.

The global predictive performance of the PINN model across the eight EAAs was evaluated (Figure 2C). The predicted concentrations generated by PINN showed an excellent correlation with the measured observations. The data points were tightly clustered along the identity line (y = x).

**Figure 3:**
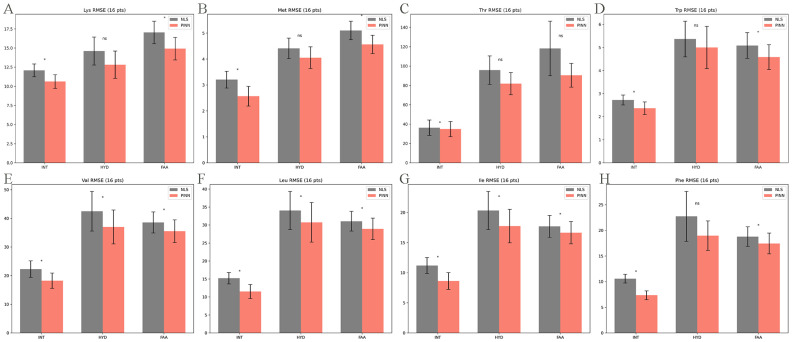
Comparison of predictive performance between NLS and PINN models across different AA profiles (n = 8). The bar charts (**A**–**H**) display the RMSE for eight EAAs: (**A**) Lys, (**B**) Met, (**C**) Thr, (**D**) Trp, (**E**) Val, (**F**) Leu, (**G**) Ile, and (**H**) Phe. Gray bars represent the traditional NLS method, while red bars represent the Physics-Informed Neural Network (PINN). Three distinct substrate conditions are compared: INT, HYD, and FAA. Data are presented as mean ± SD. Asterisks (*) denote a significant difference between groups (*p* < 0.05), and ‘ns’ indicates no significant difference.

To further investigate the consistency of the PINN framework’s superiority, we performed a granular error analysis for each of the eight EAAs under the full-sampling scenario (16 time points). As shown in Figure 3, under the INT condition, PINN demonstrated a significant advantage (*p* < 0.05) over NLS for all evaluated profiles, including Lys, Methionine (Met), Threonine (Thr), Tryptophan (Trp), Valine (Val), Leucine (Leu), Isoleucine (Ile), and Phenylalanine (Phe). Under the FAA condition, the superiority of PINN remained statistically significant for seven out of the eight AAs, with Thr being the only exception (*p* > 0.05). Under the HYD condition, PINN significantly reduced error for Val, Leu, and Ile (*p* < 0.05), while the differences for Lys, Met, Thr, Trp, and Phe did not reach statistical significance (*p* > 0.05).

### 3.2. Robust Trajectory Reconstruction Under Sparse Sampling

**Figure 4:**
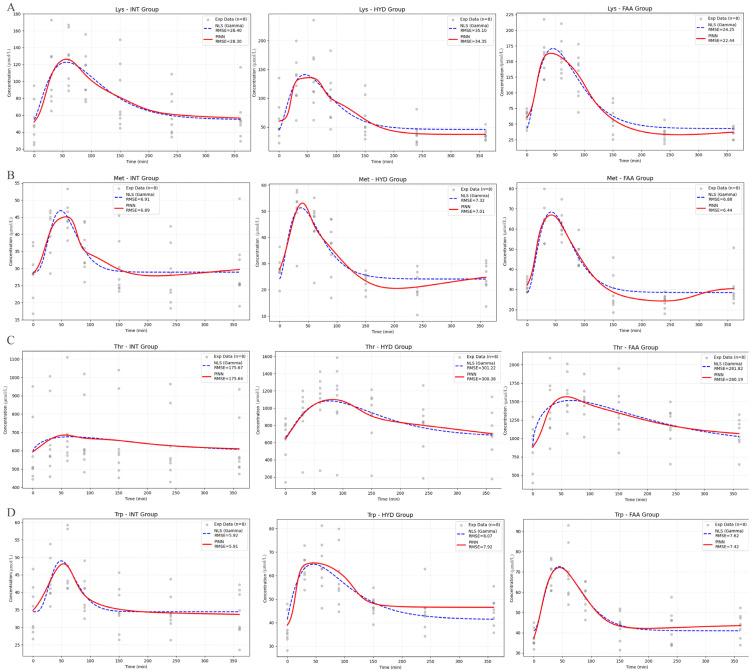
Kinetic modeling of AA absorption using PINN versus NLS approaches. Concentration-time profiles for Lys (**A**), Met (**B**), Thr (**C**), and Trp (**D**) across INT, HYD, and FAA treatment groups. Experimental data points (n = 8) are overlaid with best-fit curves obtained from the Physics-Informed Neural Network (PINN, red solid line) and the Non-linear Least Squares method (NLS, blue dashed line). The fit accuracy is indicated by the RMSE values shown in the legends.

Figure 4 shows a comparative analysis of kinetic fitting performance between PINN and NLS models across four AAs (Lys, Met, Thr, Trp, Leu, Ile, Phe). The experimental data were fitted using a traditional NLS method based on the Erlang function and the proposed PINN. Visually, both models captured the typical rapid absorption and subsequent elimination phases of AA kinetics. However, quantitative evaluation revealed that the PINN model consistently outperformed the NLS approach. As indicated by the RMSE values in each panel, PINN achieved lower prediction errors in the different treatment groups (INT, HYD, and FAA). For instance, in the Lys-FAA group, the RMSE for PINN was reduced to 22.44 compared to 24.25 for NLS. This suggests that incorporating physical laws into the neural network architecture enhances the model’s ability to generalize and capture complex non-linear dynamics in pharmacokinetic data, particularly in regions of peak concentration and metabolic clearance.

**Figure 5:**
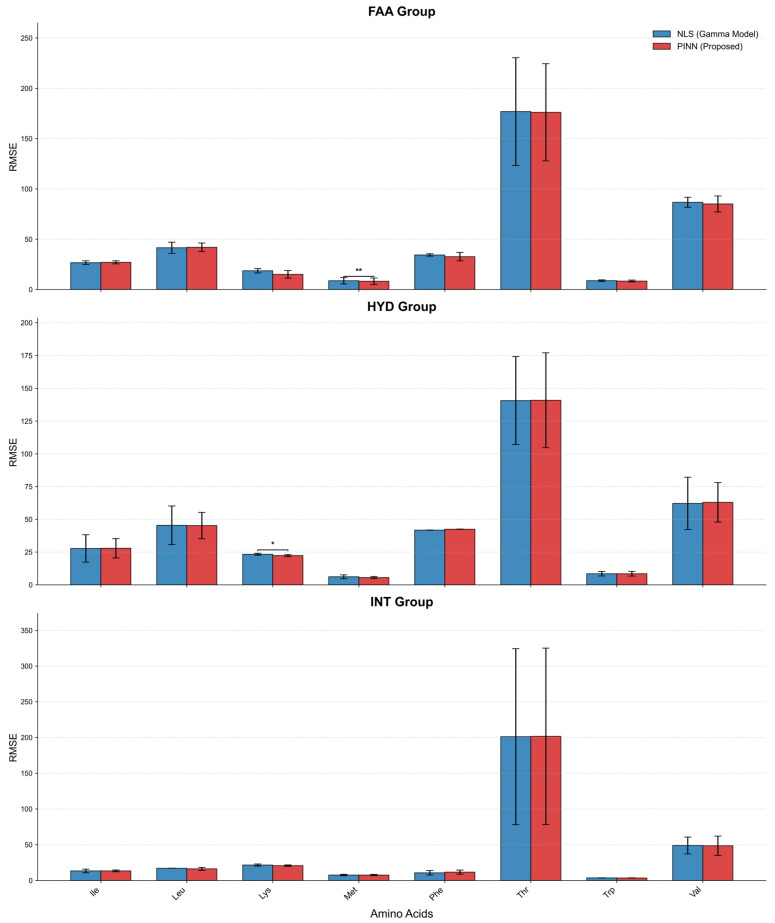
Comparative assessment of fitting errors (RMSE) between NLS and PINN models (n = 8). Bar charts displaying the RMSE for eight EAAs (Ile, Leu, Lys, Met, Phe, Thr, Trp, Val) across (**Top**) FAA, (**Middle**) HYD, and (**Bottom**) INT groups. Blue bars represent the NLS Erlang model, and red bars represent the PINN. Data are presented as mean ± SD. Asterisks indicate statistically significant differences between the two models (* *p* < 0.05, ** *p* < 0.01).

To statistically evaluate the goodness-of-fit, we compared the RMSE of the proposed PINN model against the baseline NLS Gamma model across three dietary groups (FAA, HYD, INT) and eight EAAs (Figure 5). Overall, the PINN model demonstrated robust performance, achieving RMSE values comparable to or lower than the NLS method in all test cases. Notably, the PINN model significantly outperformed the NLS approach in specific scenarios. As shown in the FAA group, the RMSE for Met was significantly reduced using PINN (*p* < 0.01). Similarly, in the HYD group, PINN exhibited a statistically significant improvement in fitting Lys kinetics (*p* < 0.05). For other AAs, such as Thr, which exhibited high variance and absolute concentration levels, both models showed similar error profiles, indicating that the PINN framework is stable across varying data magnitudes.

### 3.3. Robustness to Initial Parameter Guesses

**Figure 6:**
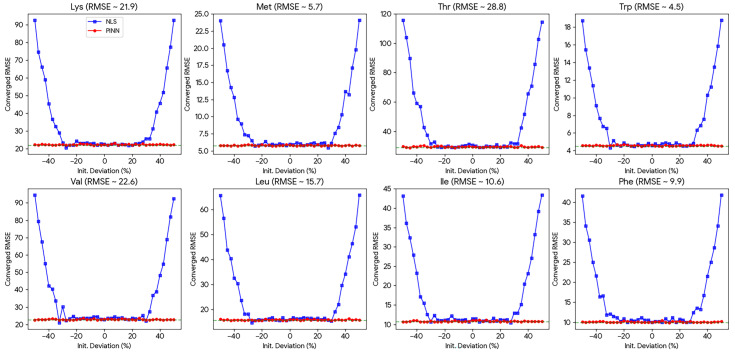
Initialization sensitivity analysis for eight essential AAs. The plots compare the convergence stability of PINN (red circles) versus traditional NLS (blue squares) under varying initial parameter perturbations (−50% to +50%). The green dashed line represents the theoretical lower bound of RMSE.

The dependence of model convergence on initial parameter guesses is visualized in Figure 6. For the eight EAAs (Lys, Met, Thr, Trp, Val, Leu, Ile, Phe), the NLS method exhibited a characteristic “U-shaped” error profile. When the initial guess deviated by more than ±25% from the true value, the NLS optimization frequently trapped in local minima, leading to an exponential surge in RMSE.

In sharp contrast, the PINN model (red circles) demonstrated exceptional stability. As shown in Figure 6, the PINN convergence trajectory remained flat and close to the ground truth across the entire perturbation range (−50% to +50%). This result confirms that the physical constraints (L_phys_) successfully guide the network to the correct solution even when prior knowledge is severely limited.

## 4. Discussion

### 4.1. PINN vs. Pure Data Fitting

The primary objective of this study was to evaluate the efficacy of PINN in modeling postprandial AA kinetics, specifically under experimentally constrained sparse sampling conditions. Our results demonstrate that the PINN framework consistently outperforms the traditional NLS regression. While both models exhibited high fidelity under dense sampling (16 points), the PINN maintained superior robustness when data availability was reduced to seven time points. Statistically, PINN achieved significantly lower RMSE values in key scenarios, such as Methionine in the FAA group and Lys in the HYD group. This indicates that the integration of the van Milgen mechanistic ODEs into the neural network’s loss function acts as a powerful regularization term. Unlike NLS, which relies solely on discrete data points and may overfit or diverge in data-sparse regions, PINN leverages the underlying physical laws to constrain the search space, ensuring biologically plausible trajectories even in the absence of dense measurements. Traditional NLS algorithms are inherently sensitive to the quality of initial parameter guesses [7]. When modeling “stiff” kinetic profiles—where concentration rises and falls abruptly—inaccurate initialization can easily cause the algorithm to diverge or become trapped in local minima, resulting in poor curve fitting. In contrast, the PINN framework circumvents this limitation by treating the governing ODEs as a regularization term within the loss function [8]. Instead of relying on manual “guesswork” to start the optimization, PINN leverages these physical constraints to automatically steer the neural network toward the optimal solution. In fact, the PINN framework is not limited to fitting EAAs but is equally applicable to Non-Essential Amino Acids (NEAAs). This versatility stems from the inherent applicability of the van Milgen equation to general AA kinetics.

### 4.2. Feasibility Analysis of Sparse Sampling Strategy via Retrospective Validation

We determined the seven specific time points (0, 30, 60, 90, 150, 240, and 360 min) based on prior studies of animal AA absorption kinetics [2,4,5,6]. Specifically, dense sampling points (30, 60, and 90 min) were included during the baseline and absorption interval (0~90 min) to characterize the rapid absorption phase and accurately locate the peak concentration and time to peak, which serve as primary indicators of nutrient bioavailability. Conversely, for the clearance and homeostasis phase (150~360 min), wider intervals (150, 240, and 360 min) were employed, as these points are sufficient to define the slower elimination rate and the return to basal homeostasis. A critical contribution of this work is the demonstration that PINN can reconstruct high-fidelity kinetic profiles from sparse data (7 time points) with accuracy comparable to dense sampling (16 time points). This finding supports the “3Rs” principle (Replacement, Reduction, Refinement) in animal experimentation. It is important to note that this validation was performed retrospectively. While our results demonstrate that PINN can reconstruct high-fidelity profiles from subsets of data, actual in vivo validation is still required to confirm these findings in practice.

### 4.3. Solving the Inverse Problem

To validate the inverse parameter identification capability of the framework, we compiled the kinetic parameters (*C_target_*, *AUC*, *λ*, *C_δ_*) derived from both PINN and NLS using the sparse seven-point dataset in Supplementary Table S1. The results demonstrate that PINN allows for the stable estimation of kinetic parameters across diverse dietary treatments (INT, HYD, FAA). It is important to note that, due to the absence of established biological reference standards for these specific kinetic parameters in the current literature, our analysis focuses on the computational stability and the model’s ability to inversely recover consistent parameters from sparse data, rather than defining absolute physiological baselines. This confirms that the PINN framework is mathematically robust in solving the inverse problem for complex postprandial dynamics.

By solving the inverse problem, PINN identifies key physiological parameters such as the fractional flow rate (λ) and the metabolic setpoint adaptation (*C_δ_*). The variations in these parameters across the INT, HYD, and FAA groups accurately reflected the known physiological differences in digestion and absorption rates. For instance, the model successfully quantified the faster absorption kinetics of the FAA group without requiring manual initial parameter guessing, which is a common bottleneck in NLS optimization. A distinct advantage of the PINN framework over standard regression is its ability to solve the inverse problem. In traditional NLS approaches, accurately estimating physiological parameters often requires providing precise initial guesses to ensure convergence, especially when data are sparse or noisy. Although this study primarily focused on the reconstruction of concentration trajectories, the underlying architecture of PINN implicitly identifies these governing parameters during the training process. By embedding the van Milgen ODEs into the loss function, the network is forced to find a solution that is not only statistically close to the data points but also physically consistent with the biological mechanisms of absorption and elimination. This approach ensures that the resulting curves—and the implicit parameters driving them—adhere to mass balance laws, reducing the risk of physiologically impossible predictions that can occur with purely data-driven interpolations.

### 4.4. Limitations and Future Directions

Despite the proposed model’s superior performance, this study has limitations. The model’s accuracy is contingent upon the validity of the underlying van Milgen ODEs; structural errors in the mechanistic model could introduce bias into the neural network predictions. Future research could explore PINN architectures with differentiated rate constants for absorption and utilization. Second, the computational cost of training PINNs is inherently higher than that of traditional NLS regression. Unlike NLS, which typically converges rapidly on standard personal computers, the optimization of neural networks involves iterative backpropagation over thousands of epochs. Furthermore, if the network architecture scales up in complexity to accommodate massive datasets, the demand for computational power will surge. This may necessitate the reliance on high-performance computing infrastructure or large-scale servers equipped with GPU acceleration, thereby increasing the operational costs of data analysis. However, when weighed against the biological costs—specifically the potential to reduce experimental sampling frequency and sampling handling stress—this computational overhead is justifiable. In the future, the application effect of this framework under different dietary conditions will be verified to determine its practical value in AA regulation.

## 5. Conclusions

Our comparative analysis demonstrates that while PINN and traditional NLS regression perform comparably under dense sampling conditions, PINN exhibits superior robustness under data-sparse scenarios (seven time points). The integration of physical laws acts as a critical regularization term, enabling the model to reconstruct high-fidelity kinetic trajectories and accurately solve the inverse problem without the need for manual parameter initialization or the risk of divergence common in NLS approaches.

## Data Availability

The data used in this study are consistent with those in the open-access repository (https://doi.org/10.57745/WHZUOQ).

## Supplementary Materials

The following supporting information can be downloaded at: https://www.mdpi.com/article/10.3390/ani16040634/s1, Table S1: Kinetic Parameters under Sparse Sampling (seven Time Points); Code: Core code.

## Abbreviations

The following abbreviations are used in this manuscript:

AA/AAs	Amino Acid(s)
NLS	Non-Linear Least Squares
PINN/PINNs	Physics-Informed Neural Network(s)
ODEs	Ordinary Differential Equations
RMSE	Root Mean Square Error
INT	Intact Protein
HYD	Hydrolyzed Protein
FAA	Free Amino Acids
EAAs	Essential Amino Acids
L-BFGS	Limited-Memory Broyden–Fletcher–Goldfarb–Shanno
Lys	Lysine
Met	Methionine
Thr	Threonine
Trp	Tryptophan
Val	Valine
Leu	Leucine
Ile	Isoleucine
Phe	Phenylalanine
NEAAs	Non-Essential Amino Acids
