# Supplementary material for: Physics-Informed Neural Networks for Modeling Postprandial Plasma Amino Acids Kinetics in Pigs
Source: Animals (Basel). 2026 Feb 16;16(4):634. doi: 10.3390/ani16040634 (PMC12937429; doi:10.3390/ani16040634)
Supplement: Supplementary file 1 [file animals-16-00634-s001.zip › Table S1.pdf]

Table S1 Kinetic Parameters under Sparse Sampling (7 Time Points)

AA	Diet	Model	Ctarget (μmol/L)	AUC (h·mol/L)	λ (h^{-1})	Cδ (μmol/L)
Ile	INT	NLS	124.59	21.23	2.51	13.61
		PINN	120.85	28.45	2.45	13.64
	HYD	NLS	83.16	178.81	2.70	34.14
		PINN	85.02	170.15	2.78	35.2
	FAA	NLS	96.91	229.95	2.80	11.08
		PINN	99.85	226.5	2.72	10.15
Leu	INT	NLS	156.41	35.61	2.73	10.05
		PINN	152.88	44.2	2.65	9.12
	HYD	NLS	115.46	281.16	2.69	61.08
		PINN	116.5	270.35	2.78	64.5
	FAA	NLS	132.39	383.92	2.73	39.32
		PINN	136.55	380.15	2.65	38.5
Lys	INT	NLS	52.5	116.63	2.13	6.73
		PINN	53.15	112.45	2.10	5.95
	HYD	NLS	60.61	148.18	2.47	-20.19
		PINN	61.85	148.3	2.50	-20.95
	FAA	NLS	61.79	202.12	2.43	-25.83
		PINN	64.5	205.15	2.44	-28.15
Met	INT	NLS	28.39	21.96	2.66	-0.03
		PINN	28.55	21.8	2.67	-0.35
	HYD	NLS	27.87	34.22	3.02	-5.09
		PINN	29.15	34.18	3.10	-6.55
	FAA	NLS	32.12	52.58	2.82	-5.26
		PINN	33.25	53.1	2.75	-6.85
Phe	INT	NLS	99.08	37.56	1.94	22.23
		PINN	97.55	39.15	1.95	23.15
	HYD	NLS	88.03	213.05	1.85	46.14
		PINN	90.5	180.5	2.01	50.25
	FAA	NLS	78.88	242.3	2.17	43.83
		PINN	81.55	245.1	2.09	42.15
Thr	INT	NLS	593.54	132.41	1.91	25.99
		PINN	590.15	135.2	1.98	29.5
	HYD	NLS	648.68	861.78	1.66	78.37
		PINN	675.5	780.5	1.70	65.25
	FAA	NLS	895.72	925.27	1.95	230.01
		PINN	915.25	925.35	1.90	210.55
Trp	INT	NLS	34.61	18.12	2.56	-1.02
		PINN	34.85	17.85	2.58	-1.15
	HYD	NLS	37.26	32.45	2.60	7.53
		PINN	38.55	30.15	2.75	6.5
	FAA	NLS	37.36	40.53	2.81	4.99
		PINN	38.25	40.85	2.72	3.85

Val	INT	NLS	461.26	78.66	2.24	-1.31
		PINN	455.5	85.15	2.22	0.55
	HYD	NLS	320.81	421.15	2.21	132.24
		PINN	324.5	380.5	2.35	140.5
Val	FAA	NLS	353.93	475.52	2.43	91.52
		PINN	358.5	470.15	2.36	94.15

INT (Intact Protein, A diet containing feather meal as the sole protein source, rep-resenting a slow-release nutrient profile); HYD (Hydrolyzed Protein, A diet containing extensively hydrolyzed feather meal (providing 82% free AAs and 18% small peptides), representing a rapid-release profile); FAA (Free Amino Acids, A synthetic diet formulated with free crystalline AAs to mimic the AA profile of the HYD diet).

Code
